# Characterization of Canine Adenovirus Type 2 Virus Infection Pattern in Canine and Human Cell Lines

**DOI:** 10.1155/2022/3658970

**Published:** 2022-12-22

**Authors:** Madison D. Hogans, Will P. Kretzschmar, Theresa A. Higgins, Atonu Chakrabortty, Rebecca L. Nance, Bruce F. Smith, Deepa Bedi, Maninder Sandey, Payal Agarwal

**Affiliations:** ^1^Scott-Ritchey Research Center, College of Veterinary Medicine, Auburn University, Auburn, USA; ^2^Department of Pathobiology, College of Veterinary Medicine, Auburn University, Auburn, USA; ^3^Biomedical Sciences, Tuskegee University, Tuskegee, AL, USA

## Abstract

Canine adenovirus type 2 (CAV2) is a nonhuman adenovirus with a known ability to infect human and canine cells. The cell surface receptors involved in CAV2 transduction are still unknown. Identification of these would provide valuable information to develop enhanced gene delivery tools and better understand CAV2 biology. CAV2 is erroneously grouped with Ad5 based on the knowledge that CAV2 may transduce using CAR. Therefore, we have evaluated CAV2 and Ad5 (CAV2GFP, Ad5G/L) infection patterns in various canine and human cell lines to determine their different tropisms. Our research demonstrates that CAV2 can successfully infect cells that Ad5 does not infect, and CAV2 infections do not correlate with CAR expression. CAV2 can infect cells that have a low or minimal expression of CAR. Our data suggest that CAV2 transduction is not dependent on the CAR receptor, and thus, it is crucial to find novel CAV2 receptors.

## 1. Introduction

Vector-mediated gene transfer is a cornerstone biomedical technique that allows investigators to understand, analyze, and modify gene functions. Efficient gene transfer is possible due to the powerful and efficient gene delivery vessels/vectors used. Adenoviral vectors are among the most commonly used tools for gene therapy.

Adenoviruses (Ad) are nonenveloped icosahedral viruses with 12 vertices that consist of fiber protein anchored in the penton base [1]. Both fiber and penton base are involved in virus binding and uptake by cells. Adenovirus transduction in cells is a two-step process [2]. In the first step, fiber binds to its receptor(s) to anchor the virus to the cells. Ad receptors for fiber from different adenoviruses include coxsackie and adenovirus receptor (CAR), CD46, sialic acid, CD80/86, heparan sulfate, and MHC class I alpha 2 domain [1]. In the second step, an amino acid motif, RGD, RGAD, or IGDD [3] present on the penton base mediates the internalization of virus particles into the cells using cell surface integrins (*α*_*v*_*β*_5_, *α*_*v*_*β*_3_, *α*_*v*_*β*_1_, *α*_5_*β*_1_, and *α*_3_*β*_1_).

Human adenovirus Ad5-based vectors are commonly used as they are diverse, and their tropism, delivery mechanism, and transgene expression can easily be modified [4]. Ad5 infects cells using CAR and cell surface integrins (*α*_*v*_*β*_5_, *α*_*v*_*β*_3_) or major histocompatibility complex class I (MHC-I) [5]. When using Ad5 vectors, cells with higher CAR expression make the best targets, but targeting cells with marginal CAR expression requires higher doses of Ad vector, leading to virus toxicity [6]. CAR is expressed on many cell types but is poorly expressed on some neuronal subtypes [7, 8], fibroblasts [9], macrophages [10], lymphocytes [11], ameloblast-like cells [12], and highly malignant cells, such as ovarian, colorectal, lung, prostate, breast, and bladder cancers [11, 13]. In some instances, CAR is expressed on cells, but transduction efficiencies are low due to a paucity of cell surface integrins (*α*_*v*_*β*_5_, *α*_*v*_*β*_3_) [14, 15]. Additional factors that may limit Ad5 transduction are cell surface MUC1, which interferes with infection by shielding integrins from interactions with the virus [16]. All these factors often lead to a failure of gene transfer, coupled, *in vivo*, with virus-induced toxicity.

Canine adenovirus type 2 (CAV2) is the most well-characterized nonhuman adenovirus with characteristics similar to Ad5 [17]. However, a comparison of the three-dimensional structure of Ad5 and CAV2 shows clear differences. CAV2 capsid is smoother than Ad5 with fewer loops in the penton base and hexon. The RGD loop in the penton base of Ad5 is involved in virus internalization. CAV2 penton does not have a conserved RGD, RGAD, IGDD, or KTKK motif [5, 18] and is likely not dependent on cell surface integrins for internalization. Interestingly, the C-terminus of CAV2 protein IX differs from Ad5 protein IX, with an antenna-like projection sticking out of the capsid [19].

CAV2 does not infect cells expressing MHC I, sialic acid, and has poor/low interaction with CD40, CD80, CD86, and CD46 [2, 5]. CAV2 vectors can transduce Ad5 refractory cells, such as neurons and ovarian cancer [20, 21]. CAV2 vectors can undergo retrograde transport in axons, thus making them good candidates to transduce neurons in specific brain areas that other vectors cannot reach [17, 22, 23]. A recombinant Ad5 vector encoding a green fluorescent protein and incorporating a CAV2 fiber knob (Ad5-CGW-CK2) increased neuronal transduction and transgene expression [8]. Recombinant Ad5 vector (Ad5Luc1-CK), encoding the luciferase gene and the fiber knob domain from CAV2, augmented gene delivery in CAR-deficient RD, CHO, U118MG, HEY, OV-3, and OV-4 cells, by up to 30-fold [5]. CAV2 transduces cells using recombinant human or murine CAR; however, CAV2 can also transduce CAR-deficient cells [5, 18]. Infection by recombinant Ad5 with CAV2 knob in CAR negative, *α*_*v*_

*β*_5_, *α*_*v*_*β*_3_ integrin positive, CHO cells indicate that CAV2 binding to cells is not dependent on CAR and that CAV2 can use an alternate receptor. Moreover, CAV2-based vectors can bind [24] but not infect CAR negative, *α*_*v*_

*β*_5_, *α*_*v*_*β*_3_ integrin positive CHO cells, making it likely that CAV2 internalization occurs through a different coreceptor than *α*_*v*_*β*_5_, *α*_*v*_*β*_3_ integrins [5].

To better understand CAV2 tropism, it is imperative to investigate CAV2 binding and infection in a wider variety of cells. While CAV2 tropism has been investigated to a limited degree in humans and mice [18], it has not been extensively pursued in canine cells, which provides the best system to understand natural CAV2 tropism. In this article, we have reported the infection patterns of CAV2 and Ad5 using replication-incompetent vectors expressing green fluorescence protein (GFP). Infections in human and canine cell lines were used to directly compare the tropism of the two adenoviruses. CAV2 infection patterns were also compared to CAR expression levels in the targeted cells.

## 2. Materials and Methods

### 2.1. Cell Culture

Canine mammary tumor cell lines CMT12, histiocytic cell line DH82, embryonic kidney cell lines FDK and MDCK, melanoma cell lines CML7 and CML10, and osteosarcoma cell lines D17 and CF11 were cultured in DMEM (Dulbecco's Modified Eagle's Medium, Corning) with penicillin (100 IU/ml, Corning), streptomycin (100 *μ*g/ml, Corning), amphotericin B (0.5 *μ*g/ml, Corning), and 10% FBS (fetal bovine serum, Sigma) [15]. Canine lymphoma cell lines 17–71 and OSW and mast cell line MPT1 were cultured in RPMI (Roswell Park Memorial Institute medium, Corning) with penicillin (100 IU/ml, Corning), streptomycin (100 *μ*g/ml, Corning), amphotericin B (0.5 *μ*g/ml, Corning), and 10% FBS (Sigma).

### 2.2. Virus Infections

The CAVGFP virus vector which encodes GFP (green fluorescence protein) under the control of the cytomegalovirus (CMV) immediate early promotor (gift of Dr. Ramone Alemany, Barcelona, Spain) and Ad5G/L virus vector, which encodes GFP (green fluorescence protein) and luciferase under the control of the cytomegalovirus (CMV) immediate early promotor (gift of Dr. David T. Curiel, St Louis, MO) was used for infections. CAVGFP and Ad5G/L virus vectors were amplified and purified using a CsCl gradient and desalted on PD-10 desalting columns (GE Healthcare) [25]. Virus particle number was measured by measuring OD260 using NanoDrop (Thermo) using the following formula: particles/ml = (OD260) × (dilution factor) × (1.1 × 10^12^) [14]. Virus infections were done at 100 multiplicities of infection (MOI; 100 virus particles/cell). 2.5 × 10^5^ cells of CMT12, DH82, FDK, MDCK, CML7, CML10, D17, and CF11 were plated in 12-well plates one day prior to virus infections. 2.5 × 10^5^ cells of 17–71, OSW, and MPT1 cell lines were plated on the same day of infections. Cells were washed with 1X PBS (phosphate buffered saline) and infected with 200 *μ*l of DMEM/RPMI (2% FBS), containing the virus. After one hour of infection, 400 *μ*l of DMEM/RPMI (10% FBS) was added to the cells. Cells were monitored at 48 hours postinfection for green fluorescence using an inverted fluorescent microscope (EVOS FL Cell Imaging System). All infections were done in triplicates.

### 2.3. Flow Cytometry

All adherent cells were harvested and washed twice with 1X PBS. All the cells were resuspended in flow wash buffer (1XPBS + 0.1% BSA; bovine serum albumin + EDTA) and analyzed for GFP expression by flow cytometry (CytoFLEX LX; Beckman Coulter and LSR-II; BD Biosciences). All experiments were performed in triplicates.

### 2.4. RNA Isolation, Primer Design, and Quantitative RT-PCR

Cell cultures were grown to 75–80% confluence, and total RNA was isolated using TRIzol reagent (Life Technologies) according to the manufacturer's instructions. The concentration of RNA was determined by absorbance at 260 nm. Canine and human conserved CAR (GenBank Accession# NM_001195845.3) (CXADR_C-H_For: CCAGAAGTTTGAGTATCACTACTC; CXADR_C-H_Rev: GATGCATCACCAGATTTGAGATC) and beta-actin (ACTB_C-H_For: GACTACCTCATGAAGATCCTCAC; ACTB_C-H_Rev: TGATGGAGTTGAAGGTAGTTTC) cDNA synthesis and amplification were performed by quantitative reverse transcriptase PCR (Q-RT-PCR) using specific primers. All qPCR reactions were conducted at 95°C for 3 minutes and then 40 cycles of 95°C for 30 seconds and 57°C for 30 seconds. The specificity of the reaction was verified by melt curve analysis. Q-RT-PCR was performed using a Bio-Rad iCycler iQ Multicolor Real-Time PCR Detection System, and assays were performed using SsoFast EvaGreen qPCR supermix (Biorad). PCR products were purified using a GeneJet gel extraction kit (Thermo) according to the manufacturer's instructions, and identity was confirmed by sequencing the amplicons (Eurofins MWG Operon).

mRNA expression was analyzed by the comparative ΔΔCt method. ΔCt values for each sample were calculated by normalizing to beta-actin as the normalization control. ΔΔCt values were calculated using the mean of ΔCt values of all samples as a reference control [26]. Finally, the relative fold CAR expression for each sample was calculated using the formula −2^−(∆∆Ct)^.

### 2.5. Statistical Analysis

Simple linear regression analysis was performed at the 95% confidence level to analyze the functional relationship between infectivity of canine adenovirus type 2 GFP and human adenovirus serotype 5G/L as determined by the percentage of cells expressing GFP by flow cytometry compared to the relative expression levels of CAR by various canine and human cell lines.

## 3. Results

### 3.1. CAVGFP and Ad5GL Virus Infections

**Figure 1:**
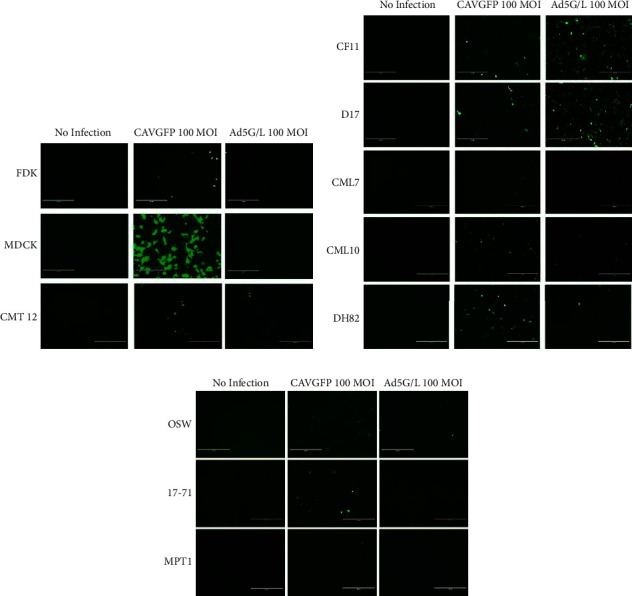
CAVGFP and Ad5G/L directed GFP expression in canine cell Lines. Cells were transduced by adenoviruses CAVGFP and Ad5G/L at 100 multiplicity of infection (MOI 100 virus particles per cell). Cells were monitored at 48 hours post-infection for green fluorescence using an inverted fluorescent microscope (EVOS FL Cell Imaging System). (a) Adherent cell lines, FDK, MDCK, and CMT12, (b) CF11, D17, CML7, CML10, and DH82, and (c) suspended cell lines, OSW, 17–71, and MPT1. This figure is a representative of three independent infection experiments.

**Figure 2:**
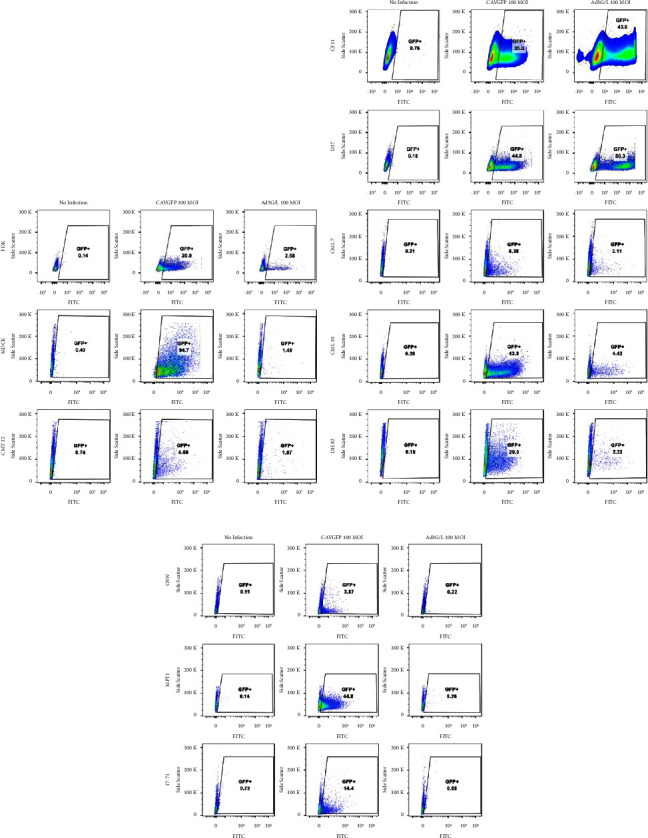
GFP Expression analysis in canine cell lines at 100 MOI by flow cytometry. Cells were transduced by Adenovirus CAV2 and Ad5G/L at 100 multiplicity of infection (MOI 100virus particles per cell). GFP expression in cells was analyzed by flow cytometry 48 hours after Ad5G/L infection. The cell population was gated for live and single cells. The GFP + cells were determined in comparison to cell with no adenovirus infection. (a) Adherent cell lines, FDK, MDCK, and CMT12, (b) CF11, D17, CML7, CML10, and DH82, and (c) suspended cell lines, OSW, 17–71, and MPT1. This figure is a representative of three independent infection experiments.

**Figure 3:**
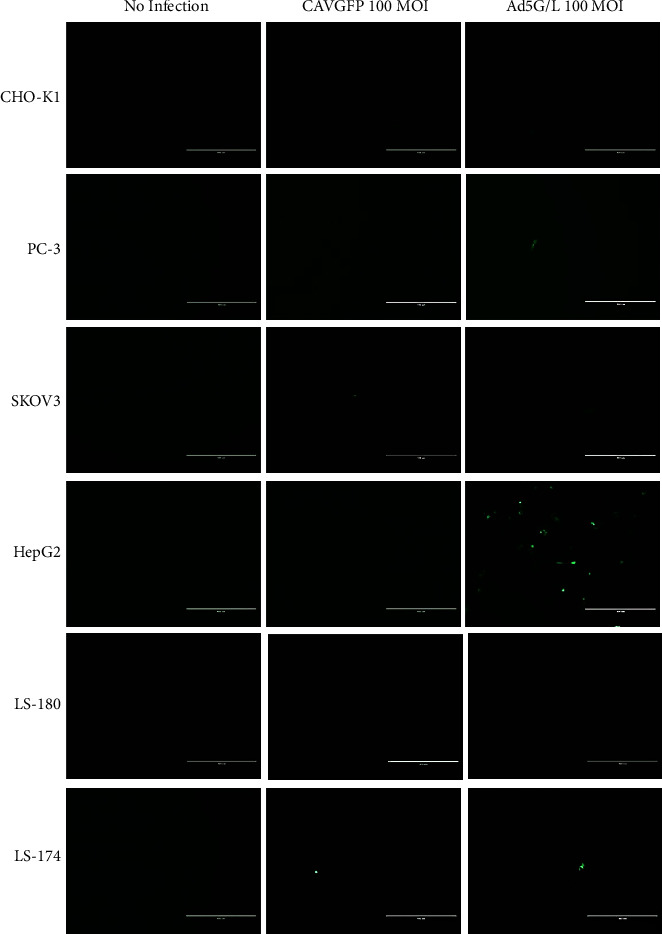
CAVGFP and Ad5G/L directed GFP expression in Chinese Hamster Ovary CHO K1 cells and human cell lines PC3, SKOV3, HepG2, LS 174, and LS 180. Cells were transduced by adenoviruses CAVGFP and Ad5G/L at 100 multiplicity of infection (MOI 100 virus particles per cell). Cells were monitored at 48 hours post infection for green fluorescence using an inverted fluorescent microscope (EVOS FL Cell Imaging System). This figure is a representative of three independent infection experiments.

**Figure 4:**
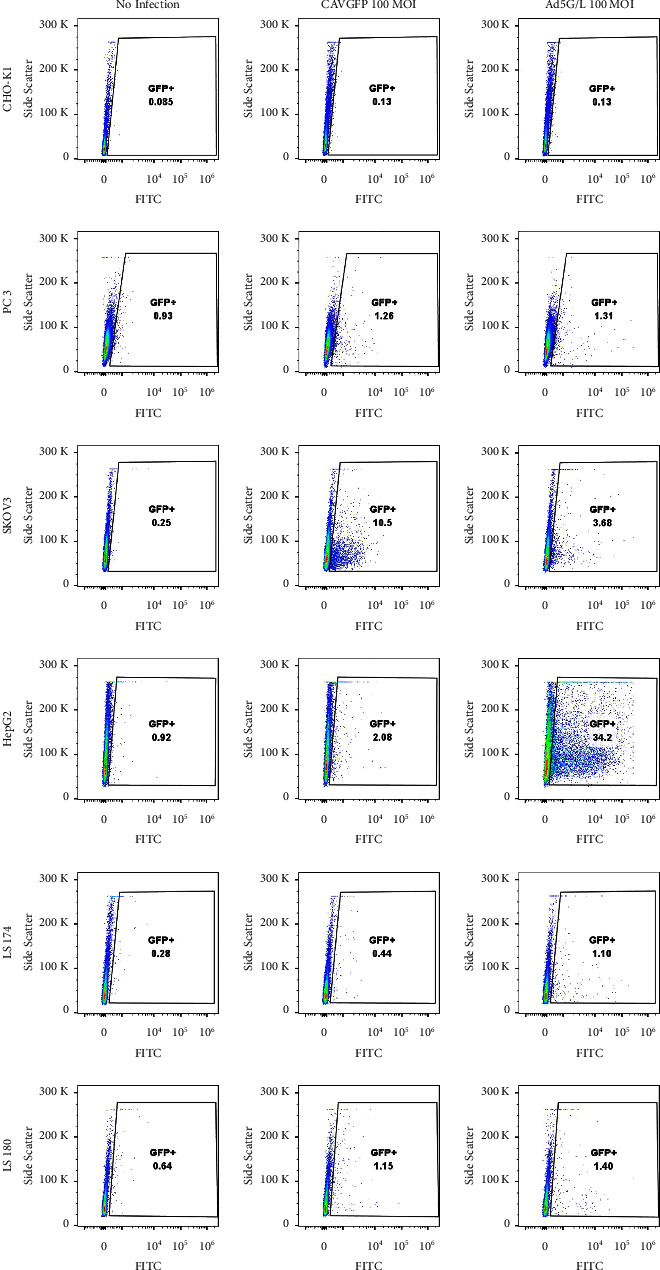
GFP Expression analysis in Chinese Hamster Ovary CHO K1 cells and human cell lines PC3, SKOV3, HepG2, LS 174, and LS 180 at 100 MOI by flow cytometry. Cells were transduced by Adenovirus CAV2 and Ad5G/L at 100 multiplicity of infection (MOI 100: 100 virus particles per cell). GFP expression in cells was analyzed by flow cytometry 48 hours after Ad5G/L infection. The cell population was gated for live and single cells. The GFP + cells were determined in comparison to cell with no adenovirus infection. This figure is a representative of three independent infection experiments.

The infection patterns of CAV2 and Ad5 were analyzed using replication-incompetent vectors expressing green fluorescence protein (GFP), CAVGFP, and Ad5G/L, respectively. In both viruses, GFP expression is under the control of the cytomegalovirus (CMV) promoter; therefore, it will show similar promoter activity levels after transduction. All canine and human cell lines (Table 1) were infected with CAVGFP and Ad5G/L at 100 MOI. The 100 MOI was selected as an average virus dose for infection to make sure that GFP + cells will contain only one virus approximately. Infection with higher MOI may cause multiple viral infections in one cell, and the virus infection is not linear. Cells were examined by fluorescent microscopy and flow cytometry (Figures 1–4; Table 1) to determine the number of cells expressing the GFP reporter gene. The level of GFP fluorescence in CAVGFP- and Ad5G/L-infected cells was compared to nontransduced cells. The infection pattern in canine adherent cell lines (FDK, MDCK, CMT12, CML7, CML10, CF11, D17, and DH82) was different between CAVGFP and Ad5G/L infections. All adherent canine cell lines expressed green fluorescence 48 hours post-CAVGFP infection (Figures 1(a), 1(b), 2(a), and 2(b)) at different levels. The percentage of the population infected ranged from highest (MDCK; 85.7%) to lowest (CMT12; 4.34%) postinfections (Table 1).

All cell lines, when infected by Ad5G/L, expressed GFP expression in a lower percentage of cells (4.22%; CML10 to 1.11%; CMT12) with the exception of CF11 (45.20%) and D17 (50.60%). More CF11 and D17 cells expressed GFP after Ad5G/L infections in comparison to CAVGFP infections (CF11; 35.90% and D17; 45.0%) (Table 1; Figure 2(b)).

The infection pattern in nonadherent canine cell lines MPT1, 17–71, and OSW was also different between CAVGFP- and Ad5G/L-infected cells (Figure 1(c)). While CAVGFP could infect all three cell lines ranging from 44.0% (MPT1) to 3.70% (OSW), Ad5G/L showed infections in a very low percentage of cells (OSW; 0.22%, 17–71; 0.89%, and MPT1; 0.23%) (Table 1; Figure 2(c)).

The infection pattern of CAVGFP and Ad5G/L is also different in human cell lines (Figures 3 and 4; Table 1). CAVGFP poorly infected most human cell lines except SKOV3 (10.90%). Similarly, only HepG2 (31.50%) was infected by Ad5G/L, and the rest of the human cell lines were poorly infected.

Both CAVGFP and Ad5G/L did not or poorly infected CHO K1 cells (Table 1; Figures 3 and 4).

### 3.2. Relative Quantification of CAR mRNA

**Figure 5:**
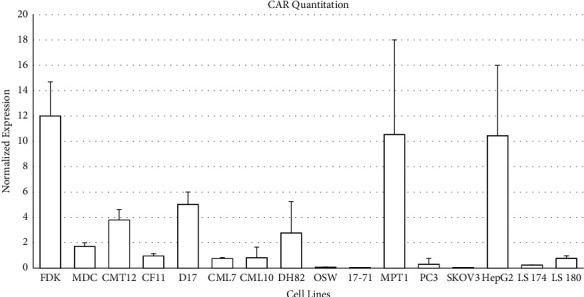
Quantitative RT-PCR analysis of CAR mRNA in canine and human cell lines using specific primers. CAR expression levels were normalized by the comparative ΔΔCt method. ΔCt values for each sample were calculated by normalizing to beta-actin as the normalization control. ΔΔCt values were calculated using the mean of ΔCt values of all samples as a reference control. The error bars represent standard deviation. The results presented here are the average of three independent qPCRs.

To better understand the mechanism for CAV2 infections and its independence of the CAR receptor, mRNA expression of CAR was compared in different cell types using Q-RT-PCR by the ΔΔCt method. CAR mRNA was expressed in almost all of the cells tested; however, the expression level varied (Figure 5). The canine cell lines FDK, CMT12, D17, MPT1, and DH82 expressed CAR at moderate to high levels. The canine cell lines MDCK, CF11, CML7, CML10, OSW, and 17–71 expressed low to nearly unmeasurable levels of CAR. Human cell lines PC3, SKOV3, and LS174 showed relatively low CAR mRNA expression while HepG2 showed moderate levels of expression.

To determine whether the infection by CAV2 and Ad5 could be statistically correlated with the expression of CAR mRNA, a simple linear regression analysis was performed. Both CAV2 and Ad5 showed no statistical correlation between infection at an MOI of 100 and expression of CAR (CAV2 *p*value: 0.2924; Ad5 *p* value: 0.7223).

## 4. Discussion

Adenoviral vectors are excellent gene therapy tools with large packaging capacities and high functional titers. Ad vectors are important therapeutically in a broad range of cells and tissues, including postmitotic cells such as neurons, and therefore are unique in that way [27]. They can be used as replication-deficient vectors, conditionally replicative vectors, conditionally targeted vectors, and vaccines to express foreign antigens and as gene therapy by altering a specific gene. However, their use is limited in cases where cells are refractory to Ad infections and lack the receptors that require for vector binding or internalization. The expression pattern of Ad-specific cell surface receptors determines the virus tropism and, therefore, the ability to use specific vectors to transduce specific cell types. It is important to identify receptors that are present on Ad refractory cells and to utilize that information to expand Ad vector tropism by introducing the corresponding binding motif in Ad fiber, hexon, or penton base to generate enhanced and more efficient Ad vectors.

To fully exploit CAV2 tropism, it is essential to define its mechanism. CAV2 vectors can transduce CAR low/negative cells [5], and while CAV2 transduction may be augmented by CAR, it is CAR independent [18]. The lack of an RGD domain and transduction of cells with blocked *α*v integrins suggests that CAV2 does not utilize a canonical internalization pathway and uses different receptors for internalization [18]. To better understand CAV2 tropism, we analyzed infection patterns of CAV2 and Ad5 using replication-incompetent vectors expressing green fluorescence protein (GFP) CAVGFP and Ad5GFP, respectively.

Results generated from our experiments confirmed that CAV2 could infect cells independent of CAR expression levels, and the pattern of CAV2 infection is significantly different from Ad5 (Figures 1–4). CAV2 was able to infect canine cells refractory to Ad5, such as FDK, MDCK, CML10, OSW, 1771, and MPT1. In contrast, canine osteosarcoma cell lines (CF11 and D17) were better infected with Ad5 than CAV2. In the limited number of human cell lines tested, CAV2 did not demonstrate any advantage over Ad5, except for a modest increase in infection levels in the ovarian cancer cell line SKOV3.

Based on these findings, CAR expression in these cells was evaluated. CAR receptor protein expressions could not be assessed in canine cell lines due to the lack of appropriately validated cross-reactive antibodies. Therefore, we examined CAR mRNA expression levels using quantitative RT-PCR. Our findings confirmed that many cells that were transduced by CAV2 have low or minimal CAR expressions, such as the canine lines MDCK, CML7, CML10, OSW, and 17–71 and the human cell line SKOV3. Ad5 infections were hampered due to low levels of CAR expression in many of the cell lines tested (MDCK, CML10, OSW, 17–71, SKOV3, PC3, LS174, and LS180). CAV2 transduction in cells (MDCK, CML7, CML10, OSW, 17–71, and SKOV3) with low or no CAR expression and Ad5's inability to infect the same cells suggest that as an alternative, as yet unidentified receptor (UIR) is utilized by CAV2 for attachment to cells.

However, FDK, CMT12, DH82, and MPT1 had higher levels of CAR levels along with higher CAV2 infections in these cells in comparison to Ad5. This phenomenon can be explained by two assumptions. First, while expressing CAR, these cells cannot be infected by Ad5 due to low levels of *αν* integrins [15]. Second, these cells express the novel CAV2 receptor along with CAR, and this receptor is responsible for infections. Alternatively, CAR may be used as an alternative receptor in the absence of the putative CAV2 receptor in these cells.

CMT12 has a moderate level of CAR expression and low infection rates by both CAV2 and Ad5. Based on our working hypothesis, this could be explained by the absence of both the CAV2 receptor and the integrins needed for Ad5 internalization. In contradistinction, CF11 with low levels of CAR, yet high levels of infection with Ad5 and moderate levels of infection with CAV2, suggests that a relatively low expression of CAR is needed for Ad5 infection.

D17 and HepG2 showed higher CAR expression levels and higher Ad5 transduction rates, as is expected according to Ad5-CAR dependence. PC3, LS174, and LS 180 have low CAR levels and, thus, low levels of Ad5 infection. The cell lines with low levels of CAV2 infection probably also have low levels of CAV2 UIR and therefore are not transduced by CAV2.

CAV2 infects OSW, 17–71, and MPT1 (Figures 2 and 3). All of these cell lines do not express integrins [15]. The ability of CAV2 to infect these cells in the absence of integrins and CAR (OSW-17-71) and the lack of RGD domain on CAV2 capsid suggest strongly that CAV2 transduction is not dependent on CAR and integrins.

Our result shows that CAV2 transductions are not dependent on and do not correlate to CAR expression in canine and human cell lines. Statistical analysis confirmed that the expression of CAR was not associated with CAVGFP transduction. Thus, the data in this manuscript suggest that CAV2 uses a receptor or receptors other than the Ad5 receptors CAR and integrins to attach to and internalize into target cells.

The ability of CAV2 to transduce a variety of human cells independent of CAR and cell surface integrin expression makes it a prime candidate for the exploration of novel host cell-surface receptors. Identification of the CAV2 fiber binding receptor(s) and coreceptors and the sites on the virus capsid responsible for binding to those receptors will allow these interactions to be exploited to enhance Ad-based gene transfer vectors. The discovery of these novel receptors would address the gaps in current gene transfer technology and will contribute in developing enhanced Ad vectors to treat more diseases than the present.

## 5. Conclusions

CAV2 transduction in cells is not dependent on CAR. The CAV2 receptor(s) and the counterpart capsid component(s) responsible for viral attachment and internalization are, as yet, unknown. To our knowledge, this is the first report to demonstrate CAV2 infections in a variety of canine cell lines. We propose that CAV2 tropism is different than Ad5, and it is essential to explore it for better gene therapy in the future.

## Figures and Tables

**Table 1:** Percentage of population infected by CAVGFP and Ad5G/L in canine and human cell lines. Cells were transduced by adenovirus CAVGFP and Ad5G/L at 100 multiplicity of infection (MOI 100 virus particles per cell). Percentage of population expressing GFP was analyzed by flow cytometry 48 hours after infection. The results presented here are the average of three independent infection experiments.

			% population	
			CAVGFP	Ad5G/L
Canine cell lines	Fetal kidney	FDK	20.8	2.77
		MDCK	85.7	1.49
	Breast tumor	CMT12	4.34	1.11
	Osteosarcoma	CF11	35.9	45.2
		D17	45	50.6
	Melanoma	CML7	6.68	2.15
		CML10	44.5	4.22
	Histiocytic tumor	DH82	20.5	2.63
	Lymphoma	OSW	3.7	0.22
		17–71	14.6	0.89
	Mast cell tumor	MPT1	44	0.23

Human cell line	Prostate cancer	PC3	1.27	1.12
	Ovarian cancer	SKOV3	10.9	3.71
	Liver cancer	HepG2	2.04	31.5
	Colon cancer	LS174	0.42	1.16
		LS180	1.15	1.42

Chinese hamster	Ovary	CHO K1	0.13	0.22

## Data Availability

All data and material that support the findings of this study are included in this manuscript.
